# A bright future for IUD use in Africa?

**DOI:** 10.9745/GHSP-D-14-00002

**Published:** 2014-02-11

## Abstract

High uptake of IUDs under the mobile outreach service delivery model in Kenya bodes well for IUDs in sub-Saharan Africa, if delivered with good access and quality.

Intrauterine devices (IUDs) have many positive attributes for clients, including high effectiveness and long duration of effectiveness. They are used very widely globally, yet not in Africa.

A number of factors contribute to this low use, but the perspective of providers is one major cause. Some providers have incorrect beliefs about high health risks associated with IUDs. Also, while IUD insertion is not terribly difficult compared with many medical procedures, provision of IUDs takes considerable wherewithal: competence, self-confidence, supplies and equipment, and, most importantly, sufficient time. In the context of a busy clinic that often provides a multitude of maternal and child health and other services, it is much easier for a provider to give an injection or a package of oral contraceptives than to interrupt the rhythm of work flow to provide counseling and insertion of an IUD. Low provider motivation and support reinforce low client demand. And even when providers are well-trained, low frequency of provision leads to a downward spiral of lost confidence and skill.

Enter the “dedicated provider.” Such providers are well-trained and well-supplied, are mentally oriented to take the time to provide IUDs, and provide them with a high enough frequency so as to have high confidence and proficiency. But does deploying IUDs in such a fashion, such as through the mobile outreach model employed by Marie Stopes and others, translate into high popularity in sub-Saharan Africa?

In this issue of GHSP, David Hubacher et al.,^1^ provide good evidence that this can indeed be the case, in the context of mobile outreach for both IUDs and implants by Marie Stopes Kenya. The authors' main purpose was to assess what effect might result from simple introduction of a discrete quantity of a newer IUD that releases the progestin levonorgestrel, called the levonorgestrel intrauterine system (LNG IUS). The findings are pretty modest. Providers tended to like the LNG IUS, and there was perhaps a small uptick in overall IUD use. However, the most remarkable finding was that the overall IUD (predominantly copper IUD) share of all mobile outreach provision was over 40%! And the rate of IUD provision for the mobile outreach teams combined was about 40,000 per year.

We already know that implants are extremely popular,^2^^,^^3^ but at least in this context, IUDs too can be very popular. In addition to accessible and quality service, it is likely that provision of IUDs over a long period of time by Marie Stopes Kenya has also built up a clientele of satisfied users, so that the positive attributes of IUDs have become more widely known. This bodes well for IUD popularity in Africa.

Mobile service delivery is one (albeit a potent one) service delivery approach. Others include postpartum and postabortion provision, social franchising, and fixed-facility services. It's approaches like these that can build a large contingent of satisfied IUD users, who will contribute a substantial share to the FP2020 goal of reaching 120 million new family planning clients by 2020. –*Global Health: Science and Practice*
